# Longitudinal evaluation of the effects of illness perceptions and beliefs about cardiac rehabilitation on quality of life of patients with coronary artery disease and their caregivers

**DOI:** 10.1186/s12955-020-01405-0

**Published:** 2020-05-27

**Authors:** Patricia Thomson, Neil J. Angus, Federico Andreis, Gordon F. Rushworth, Andrea R. Mohan, Misook L. Chung, Stephen J. Leslie

**Affiliations:** 1 Faculty of Health Sciences and Sport, University of Stirling, Stirling, FK94LA Scotland, UK; 2 School of Health, Social Care and Life Sciences, University of the Highlands and Islands, Centre for Health Science, Old Perth Road, Inverness, IV2 3JH Scotland, UK; 3 Highland Pharmacy Education & Research Centre, Centre for Health Science, Old Perth Road, Inverness, IV2 3JH Scotland, UK; 4 School of Nursing and Health Sciences, University of Dundee, Dundee, DD1 4HN Scotland, UK; 5 College of Nursing, University of Kentucky, Lexington, KY 40506 USA; 6 Cardiac Unit, Raigmore Hospital, NHS Highland, Old Perth Road, Inverness, IV2 3UJ Scotland, UK

**Keywords:** Cardiac rehabilitation, Illness perceptions, Beliefs about cardiac rehabilitation, Physical health, Mental health, The actor-partner interdependence model

## Abstract

**Background:**

Patients’ negative illness perceptions and beliefs about cardiac rehabilitation (CR) can influence uptake and adherence to CR. Little is known about the interpartner influence of these antecedent variables on quality of life of patients with coronary artery disease (CAD) and their family caregivers. The aims of the study were: 1) to assess differences in illness perceptions, beliefs about CR and quality of life between patients with CAD and their family caregivers upon entry to a CR programme and at 6 months follow-up; and 2) to examine whether patients’ and caregivers’ perceptions of the patient’s illness and beliefs about CR at baseline predict their own and their partner’s quality of life at 6 months.

**Methods:**

In this longitudinal study of 40 patient-caregiver dyads from one CR service, patients completed the Brief Illness Perception Questionnaire and Beliefs about Cardiac Rehabilitation Questionnaire at baseline and 6 months; and caregivers completed these questionnaires based on their views about the patient’s illness and CR. The Short-Form 12 Health Survey was used to assess patients’ and caregivers’ perceived health status. Dyadic data were analysed using the Actor–Partner Interdependence Model.

**Results:**

Most patients (70%) were men, mean age 62.45 years; and most caregivers (70%) were women, mean age 59.55 years. Caregivers were more concerned about the patient’s illness than the patients themselves; although they had similar scores for beliefs about CR. Patients had poorer physical health than caregivers, but their level of mental health was similar. Caregivers’ poorer mental health at 6 months was predicted by the patient’s perceptions of timeline and illness concern (i.e. *partner effects*). Patient’s and caregiver’s illness perceptions and beliefs about CR were associated with their own physical and mental health at 6 months (i.e. *actor effects*).

**Conclusions:**

Overall, the patients and caregivers had similar scores for illness perceptions and beliefs about CR. The *actor* and *partner effect* results indicate a need to focus on specific illness perceptions and beliefs about CR, targeting both the individual and the dyad, early in the rehabilitation process to help improve patients and caregivers physical and mental health (outcomes).

## Background

Cardiac rehabilitation (CR) is commonly offered after a cardiac event or coronary intervention. It promotes the affected individual’s recovery and adjustment and aims to reduce the likelihood of further cardiac illness and improve prognosis [1, 2]. Participation in CR is known to impact positively on patient outcomes and reduce subsequent morbidity and mortality [3, 4]. Comprehensive CR includes a combination of health education, supervised exercise and psychosocial support [5]. Despite the established benefits of CR, the engagement rates to the CR remain sub-optimal with a recent estimate that only 50% of all eligible patients, on average, participate in the United Kingdom [6].

A range of physical barriers to CR attendance have previously been identified and extensively investigated [3, 7, 8]. These include the distance from CR provision, age, gender, ethnicity, pre-existing low levels of physical activity and early return to work following a cardiac event. Relatively less is known about psychosocial factors that impact the outcomes of patients in CR although illness perceptions appear to be an important variable [9].

Patients’ negative illness perceptions can act as a significant barrier to uptake of and adherence to CR [5, 10]. These perceptions represent an individual’s beliefs about their illness and are known to influence coping and response to perceived health threats. Crucially such perceptions may impact subsequent health behaviours and outcomes [11], a phenomenon previously linked to several long-term conditions following a medical diagnosis [12–14]. Furthermore, illness perceptions may change during CR and over time and this can also impact on future quality of life [15].

In addition to patients’ illness perceptions those of partners and caregivers ought to be carefully considered given their inter-dependence and the possibility that the reaction of caregivers may serve to enhance or impede a patient’s adjustment to illness [16, 17].

Positive caregiver support can help to facilitate recovery and adjustment following an acute cardiac event [18], and researchers have long advocated the merit of evaluating inter-partner influences over time [19, 20], but to date most studies have been conducted in cancer care or with older adults [21–23]. Although there have been some cross-sectional dyadic studies in cardiac populations [20–24], there is currently a paucity of longitudinal research examining how caregivers perceive cardiac patients’ illness and rehabilitation and how this impacts on both parties at the dyadic level. Thus, simultaneous exploration of patient and caregiver perceptions, informed by the study of patient-caregiver dyads, is both necessary and justified. This approach offers scope to develop an enhanced understanding of inter-partner influences on recovery over time as well as a potential basis for improving patient and caregiver adjustment to cardiac illness. This knowledge can help inform future interventions aimed at supporting patient and caregiver adjustment to cardiac illness and thereby enhance the physical and mental health of both. This study aimed: 1) to assess differences in illness perceptions, beliefs about CR and quality of life between patients with coronary artery disease (CAD) and their family caregivers upon entry to a CR programme and at 6 months follow-up; 2) to examine whether patients’ and caregivers’ perceptions of the patient’s illness and beliefs about CR at baseline predict their own and their partner’s quality of life at 6 months.

## Method

### Study design, setting and sample

This was a longitudinal study of patients with CAD and their family caregivers who were followed for 6 months. Data were collected using a convenience sample of patients with CAD attending one hospital-based CR service in one NHS Board in northern Scotland between 2014 and 2015. The number of study subjects was determined by practical considerations; further details on the sample size and response rates are given in the results section. Eligible patients were aged 45 years or over, had a confirmed medical diagnosis of CAD and were on stable doses of cardiac prevention medication. We recruited spouses and partners of patients (hereafter referred to as family caregivers or caregivers) who lived in the same household as the patient and were identified by them as their primary carer. Patients and caregivers were excluded if there were any major co-morbidities such as stroke or cancer, or psychological or communication problems that affect their ability to provide informed consent.

### Recruitment and data collection

Patients were recruited on their first visit to the CR program. Caregivers were recruited via patients. Study Information and consent forms were distributed by CR specialists following the inclusion and exclusion criteria. After receipt of the signed consent forms, the researcher (PT) posted questionnaire packs to the participant’s home address or provided a link to the Bristol on-line survey for completion, depending on their preferred method. The patient-caregiver pair were asked to complete the questionnaires without discussing their answers with each other. Completed questionnaires were returned to the researcher by post or email. A reminder letter was sent after 2 weeks. After 6 months the participants were contacted again to complete the follow-up questionnaires.

### Ethical approval

This study was approved by the University of Stirling Ethics and Research Committee and the National Research and Ethics Committee (NRES), North of Scotland (Rec ref. 13/NS/0152 (IRAS project ID: 133236). A written consent was obtained from all participants in the study i.e. both patients and caregivers.

### Measures

#### Illness perceptions

Patients’ illness perceptions were assessed using the Brief Illness Perception Questionnaire (B-IPQ) [25], which consists of eight items: consequences (i.e. how much does your illness affect your life?); timeline (i.e. how long do you think your illness will continue?); personal control (i.e. how much control do you feel you have over your illness?); treatment control (i.e. how much do you think your treatment can help your illness?); identity (i.e. how much do you experience symptoms from your illness?); illness concern (i.e. how concerned are you about your illness?); coherence (i.e. how well do you understand your illness?)) and emotional representation (i.e. how much does your illness affect you emotionally e.g. does it make you angry, scared, upset or depressed?). Caregivers’ perceptions of the patient’s illness were assessed by them answering each question according to their view about their partner’s (i.e. the patient’s) illness (CAD.) Each item on the B-IPQ is scored from 0 to 10. Increases in item scores represent linear increases in each of the dimension measured [25], i.e. effect of illness on life, duration of illness, perceived control over illness, perceived efficacy of treatment, symptom experience, level of illness concern, understanding of illness and emotional impact of illness. The cumulative score for items 1–8 gives a score range of 0 to 80. In order to compute the overall score, items 3, 4, and 7 were reverse coded. A higher total score reflects a more threatening (negative) view of the illness [25]. The B-IPQ also has a causal representation item (item 9), which requires an open-ended response (not reported in this paper). The B-IPQ has shown good validity [11, 25], in research with cardiac patients [26, 27], and spouses [28]. A slight modification was made to the B-IPQ to fit the context relevant to caregivers [28]. This involved rewording the introduction to indicate to the caregivers how to answer the questions appropriately with respect to the patient’s illness and not themselves. Cronbach’s alpha for the B-IPQ (total score) was 0.75 for patients and 0.65 for caregivers.

#### Beliefs about cardiac rehabilitation

Patients’ and caregivers’ beliefs about the patient’s CR were assessed using the 13 item Beliefs about Cardiac Rehabilitation Questionnaire (BCR-Q) [29], a self-administered tool containing four sub-scales: perceived necessity (5 items); concerns about exercise (3 items); practical barriers (3 items); and perceived suitability (2 items). All items were rated on a 5-point Likert scale, from 1 (= strongly disagree) to 5 (= strongly agree), with the exception of one item on the necessity scale, that is ‘some aspects of the CR programme are unnecessary for me’, which was reversed scored. For each sub-scale, the scores were summed and means obtained for: necessity (range 9–25); concerns about exercise (range 3–15); practical barriers (range 3–15); and perceived suitability (range 2–10). Higher scores by subscale indicate the individual is more likely to agree that CR is necessary; has more concerns about exercise; perceives more practical barriers; and that CR is probably more suitable for a younger, more active person [29]. The BCR-Q has been shown to be a valid and reliable measure of beliefs about CR [29, 30]. In this study, a modification was made to the BCR-Q to fit the context relevant to caregivers, which involved rewording the introduction to indicate to them how to answer the questions appropriately with respect to the patient’s CR. Cronbach’s alpha for the 4 sub-domains of the BCR-Q were 0.68–0.76 for patients and 0.62–0.76 for caregivers.

#### Quality of life

Patients’ and caregivers’ quality of life was assessed using the Medical Outcomes Short-Form 12 (version 2) Health Survey (SF-12v2) [31], which is composed of 12 items; which are aggregated into two summary components: the physical component score (PCS) and mental component score (MCS). Rated items reflect what the individual could do functionally, how they felt and how they evaluated their own health status. Quality of life was regarded as a multi-dimensional construct to include subjective evaluation of the individual’s physical and mental health and social functioning. The SF-12v2 scores were calculated following the norm-based scoring algorithm, using weights derived from confirmatory factor analysis [32]. The measure has demonstrated good validity and reliability [31, 33, 34], and it has been used in studies of cardiac patients [2, 35–39], and patient-caregiver dyads [40–42]. Cronbach’s alpha for the PCS was 0.77 for patients and 0.89 for caregivers; and the MCS was 0.81 for patients and 0.91 for caregivers.

#### Socio-demographic and clinical characteristics

We collected socio-demographic and clinical data to describe the sample characteristics. Occupation was identified by the Office of National Statistics (ONS 1998). The Carstairs index [39], provided social deprivation categories based on the postcode region in Scotland. Scores range from 1 to 7, with higher categories indicating greater deprivation (i.e. lower socio-economic status). Diagnosis, revascularisation, left ventricular ejection fraction, cardiac history, co-morbidity (i.e. hypertension, diabetes), other cardiovascular disease risk factors and current medications were identified from the patient’s clinical records.

#### Statistical analysis

Paired sample t-tests were used to examine differences in patients’ illness perceptions (and caregivers’ perceptions of the patient’s illness), beliefs about CR and quality of life, compared within two dyad members at baseline and 6 months. For the second specific aim, longitudinal multi-level dyadic regression modelling, the Actor-Partner Inter-dependence Model (APIM) for distinguishable dyads was used [43, 44]. *Actor effects* refers to the impact of an individual’s characteristics i.e., the patient’s illness perceptions (and caregiver’s perceptions of the patient’s illness) and beliefs about CR at baseline on their own quality of life at 6 months, while we controlled for individuals’ quality of life at baseline. *Partner effects* refers to the impact of an individual’s characteristics i.e., then patient’s illness perceptions (and caregiver’s perceptions of the patient’s illness) and beliefs about CR at baseline on his or her partner’s quality of life at 6 months while controlling the quality of life at baseline.

For the dyadic analysis, all data were restructured to a pairwise dyadic data set. Grand-mean centred scores were created that were standardized using *z* scores to obtain unstandardized and standardized regression coefficients for the *actor* and *partner effects* [43]. Thirteen separate APIM models were computed for the physical component score (PCS) and 13 APIM models were computed for the mental component score (MCS). Physical health (PCS) and mental health (MCS) at 6 months were regressed on baseline illness perceptions (B-IPQ, total score and 8 individual items) and beliefs about CR (4 sub-domains), controlling for baseline physical health (PCS) and mental health (MCS). All analyses were performed using SPSS version 21.0 for Windows, with *p* < 0.05 indicating statistical significance.

## Results

### Recruitment

Fifty-six patient-caregiver dyads consented to participate in the study and completed the questionnaires at baseline. At 6 months follow-up, 40 (71%) of these dyads completed the questionnaires so that only 40 dyads (= 80 individuals) were included in this study.

### Characteristics of the participants

Table 1 presents the socio-demographic and clinical characteristics of the participants. Most patients were men (70%) and the mean age was 62.45 years (SD = 7.84). Most caregivers were women (70%) and the mean age was 59.55 years (SD = 10.05). More than half the patients (52.5%) had a non-ST elevation myocardial infarction (NSTEMI) and 22.5% had a diagnosis of ST elevation myocardial infarction (STEMI) (Table 1). Following their cardiac event and joint assessment by the nurse and physiotherapist patients commenced their exercise based CR classes between 3 and 5 weeks post discharge, 6 weeks if post-surgical patients.

**Table 1:** Patients and family caregivers characteristics (*n* = 40 dyads)

Characteristics	Patients (*n* = 40)	Caregivers (*n* = 40)
Age, years (mean, range)	62.45 (45–78)	59.55 (36–79)
Males	28 (70%)	12 (30%)
Marital status		
Married	37 (92.5)	–
Co-habilitating	3 (7.5%)	–
Employment		
Employed	20 (50%)	24 (60%)
Unemployed or retired	20 (50%)	16 (40%)
Education, years (median, range)	14.0 (7–30)	14.7 (10–30)
Social deprivation (SIMD)		
SIMD 1–2	10 (25%)	–
SIMD 3–5, out of area	30 (75%)	–
Diagnosis		
STEMI	9 (22.5%)	–
NSTEMI	21 (52.5%)	–
Unstable angina	5 (12.5%)	–
Stable angina	5 (12.5%)	–
Revascularisation		
Thrombolysis	2 (5%)	–
PCI	32 (80%)	–
CABG	1 (2.5%)	–
Left ventricular ejection fraction		
> 50%	21 (52.5%)	–
30–49% (mild-moderate impairment)	17 (42.5%)	–
< 29% (severe impairment), or missing	2 (5%)	–
Cardiac history		
PCI	5 (12.5%)	–
CABG	3 (7.5%)	–
Myocardial infarction	4 (10%)	–
Co-morbid conditions		
Hypertension	23 (57.5%)	–
Diabetes mellitus	2 (5%)	–
Other CVD risk factors		
Smoking	16 (40%)	–
Missing data	10 (25%)	–
Hypercholesterolaemia	21 (52.5%)	–
Missing data	2 (5%)	–
Medications		
ACE/ARB	22/3 (62.5%)	–
Beta blocker	29 (72.5%)	–
Diuretics	2 (5%)	–
Antidepressants	6 (15%)	–

*SIMD* Scottish Index of Multiple Deprivation, *STEMI* ST elevation myocardial infarction, *NSTEMI* non-ST elevation myocardial infarction, *ACE* angiotensin converting enzyme inhibitor, *ARB* angiotensin receptor blocker

Thirty patients (75%) and twenty-four caregivers (60%) completed questionnaires via the Bristol on-line survey and the remainder completed paper copies, as their preferred method.

### Differences in perceptions of illness, beliefs about cardiac rehabilitation and quality of life

Table 2 shows the patients’ and caregivers’ scores for illness perceptions, beliefs about CR and physical and mental health at time-point 1 (baseline) and time-point 2 (6 months). Caregivers had a significantly higher total score for B-IPQ than patients at 6 months follow-up (49.15 vs 29.43, *p* < 0.001), indicating they had a more threatening (negative) view of the patient’s illness. However, among 8 items of perceptions of the patient’s illness, caregivers had higher scores only for illness concerns (B-IPQ, individual item) than patients at baseline (7.43 vs 5.65, p 0.003) and 6 months (6.33 vs 4.95, p 0.01). This indicated they were more concerned about the patient’s illness than the patients themselves (Table 2). The patients and caregivers BCR-Q scores for necessity, concerns about exercise, practical barriers and suitability were similar at baseline and 6 months follow-up (Table 2). Given the possible range of scores, concerns about exercise, barriers to CR and perceived suitability were slightly lower (i.e. less positive), but the necessity scores were higher (i.e. more positive). Table 2 presents the physical health (PCS) and mental health (MCS) scores. The patients’ physical health (PCS) was low at baseline and 6 months, indicating poor physical health; both the patients and caregivers had a sub-optimal level of mental health at baseline and 6 months. Additional information on illness perceptions, beliefs about CR and physical and mental health are shown in Table 2.

**Table 2:** Comparison of perceptions of illness, beliefs about cardiac rehabilitation and quality of life (40 dyads)

Illness perceptions:	Time point	Patients Means (SD)	Caregivers Means (SD)	*p*-value	Beliefs about CR:	Time point	Patients Means (SD)	Caregivers Means (SD)	p-value
Consequences	TP1 TP2	4.53 (2.5) 3.28 (2.6)	5.45 (2.8) 3.85 (3.1)	0.095 0.297	Necessity	TP1 TP2	18.65 (2.4) 18.65 (3.8)	18.53 (2.5) 18.78 (4.2)	0.814 0.887
Timeline	TP1 TP2	7.18 (3.4) 7.73 (3.1)	6.65 (2.9) 7.88 (3.2)	0.390 0.838	Concerns exercise	TP1 TP2	5.85 (2.5) 5.85 (2.7)	5.50 (2.3) 5.63 (2.3)	0.486 0.649
Personal control	TP1 TP2	6.65 (2.1) 6.75 (2.3)	6.38 (2.7) 6.38 (2.7)	0.569 0.469	Practical barriers	TP1 TP2	4.58 (1.9) 5.05 (2.1)	4.38 (1.7) 4.80 (2.4)	0.520 0.648
Treatment control	TP1 TP2	8.73 (1.5) 8.25 (1.8)	8.85 (1.4) 8.38 (2.1)	0.644 0.739	Suitability	TP1 TP2	3.65 (1.9) 3.53 (1.5)	3.70 (1.6) 3.85 (1.7)	0.900 0.373
Identity	TP1 TP2	3.03 (2.4) 2.73 (2.3	3.98 (2.4) 3.08 (2.5)	0.082 0.406	**Quality of life (SF-12)**	Time point	Patients Means (SD)	Caregivers Means (SD)	p-value
Illness concern	TP1 TP2	5.65 (2.9) 4.95 (2.9)	7.43 (2.3) 6.33 (2.9)	0.003** 0.010**	Physical health (PCS)	TP1 TP2	47.03 (8.2) 48.50 (9.5)	53.55 (9.4) 54.20 (6.9)	0.002** 0.001***
Coherence	TP1 TP2	8.53 (1.5) 8.23 (1.7)	8.65 (1.8) 8.50 (1.9)	0.677 0.501	Mental health (MCS)	TP1 TP2	47.58 (8.8) 48.68 (10.1)	44.29 (14.6) 47.14 (11.3)	0.268 0.576
Emotional response	TP1 TP2	3.98 (2.9) 3.98 (2.9)	5.10 (3.1) 4.78 (3.1)	0.134 0.269					
B-IPQ (total score)	TP1 TP2	30.48 (11.9) 29.43 (12.8)	34.73 (9.9) 49.15 (9.9)	0.077 0.001***					

*B-IPQ* Brief Illness Perception Questionnaire, *PCS* physical component score, *MCS* mental component score, *TP1* time-point 1 (baseline), *TP2* time-point 2 (6 months), *SD* standard deviation; *p* < 0.05*; *p* < 0.01**, *P* < 0.001***

### Illness perceptions/beliefs about CR as predictors of quality of life in dyadic relationships

**Fig. 1:**
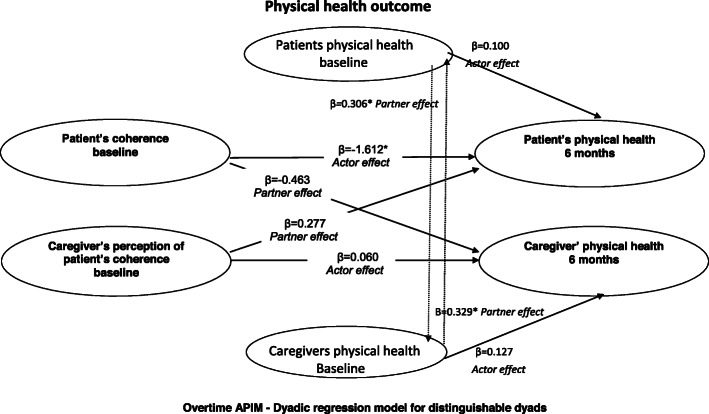
Results for the *actor* and *partner* effects of patient coherence and caregiver’s perception of patient’s coherence on the patient’s and caregiver’s physical health (SF-12) at 6 months; APIM: Actor-Partner Interdependence Model. *p < 0.05

Results for the predictors of physical health (outcome) in patient-caregiver dyads are presented in Table 3**.** For illness perceptions (B-IPQ), the patient’s higher scores for coherence i.e. how well they felt they understand their illness at baseline significantly predicted their poorer physical health at 6 months (i.e. *actor effect)* (Table 3, Fig. 1). Also, the patient’s higher scores for practical barriers to CR at baseline significantly predicted their poorer physical health at 6 months (i.e. *actor effect*). There were no other statistically significant *actor effects* of baseline illness perceptions (B-IPQ) on the physical health of patients or caregivers at 6 months follow-up; and also no other *actor* or *partner effects* of baseline beliefs about CR (BCR-Q) on the physical health of patients or caregivers at 6 months (Table 3).

**Table 3:** Illness perceptions and beliefs about cardiac rehabilitation as predictors of physical health outcome (APIM)

Effect	Patients			Caregivers			Effect	Patients			Caregivers		
**PCS outcome**	Beta	*t*	*p*	Beta	*t*	*p*	**PCS outcome**	Beta	*t*	*p*	Beta	*t*	*p*
**Illness perceptions**							**Illness perceptions**						
Total score:							Consequences:						
Actor effect (B-IPQ)	− 0.100	− 0.941	0.350	0.067	0.636	0.527	Actor effect (B-IPQ)	− 0.341	− 0.708	0.481	0.109	0.226	0.822
Partner effect (B-IPQ)	0.008	0.066	0.948	− 0.008	− 0.067	0.947	Partner effect (B-IPQ)	− 0.339	− 0.784	0.435	− 0.076	− 0.177	0.860
Actor effect (PCS)	0.070	0.431	0.668	0.171	1.056	0.294	Actor effect (PCS)	0.086	0.545	0.587	0.133	0.841	0.403
Partner effect (PCS)	0.327	2.439	0.017*	0.323	2.407	0.018*	Partner effect (PCS)	0.344	2.499	0.015*	0.344	2.502	0.014*
Timeline:							Personal control:						
Actor effect (B-IPQ)	− 0.004	−0.014	0.989	− 0.016	− 0.045	0.964	Actor effect (B-IPQ)	− 1.080	− 1.912	0.059	0.634	1.123	0.265
Partner effect (B-IPQ)	0.272	0.622	0.536	0.410	0.940	0.350	Partner effect (B-IPQ)	0.103	0.233	0.816	0.447	1.009	0.316
Actor effect (PCS)	0.130	0.860	0.392	0.144	0.950	0.345	Actor effect (PCS)	0.111	0.754	0.453	0.139	0.944	0.348
Partner effect (PCS)	0.355	2.578	0.012*	0.358	2.601	0.011*	Partner effect (PCS)	0.355	2.684	0.009*	0.317	2.403	0.019*
Treatment control:							Identity:						
Actor effect (B-IPQ)	0.610	0.742	0.460	0.143	0.174	0.862	Actor effect (B-IPQ)	− 0.933	− 1.874	0.065	− 0.534	− 1.073	0.286
Partner effect (B-IPQ)	− 0.247	−0.274	0.785	− 0.059	− 0.066	0.948	Partner effect (B-IPQ)	0.076	0.145	0.885	0.125	0.238	0.813
Actor effect (PCS)	0.123	0.807	0.422	0.140	0.920	0.360	Actor effect (PCS)	0.095	0.601	0.549	0.123	0.780	0.438
Partner effect (PCS)	0.329	2.464	0.016*	0.333	2.495	0.015*	Partner effect (PCS)	0.364	2.687	0.009*	0.379	2.792	0.007*
Illness concern:							Coherence:						
Actor effect (B-IPQ)	0.282	0.722	0.473	−0.750	− 1.913	0.059	Actor effect (B-IPQ)	− 1.612	− 2.011	0.048*	0.060	0.076	0.940
Partner effect (B-IPQ)	− 0.241	− 0.450	0.654	0.158	0.295	0.769	Partner effect (B-IPQ)	− 0.463	− 0.638	0.525	0.277	0.382	0.703
Actor effect (PCS)	0.144	0.911	0.365	0.088	0.559	0.578	Actor effect (PCS)	0.100	0.675	0.502	0.127	0.856	0.395
Partner effect (PCS)	0.361	2.779	0.007*	0.304	2.343	0.022*	Partner effect (PCS)	0.306	2 .293	0.024*	0.329	2.468	0.016*
Emotional response:													
Actor effect (B-IPQ)	0.123	0.307	0.759	−0.602	−1.498	0.138							
Partner effect (B-IPQ)	−0.116	− 0.282	0.778	− 0.326	− 0.794	0.429							
Actor effect (PCS)	0.072	0.467	0.641	0.138	0.871	0.386							
Partner effect (PCS)	0.350	2.674	0.009*	0.306	2.337	0.022*							
**Beliefs about CR**							**Beliefs about CR**						
Necessity:							Exercise concern:						
Actor effect (BCR-Q)	− 0.256	− 0.487	0.628	− 0.197	−0.375	0.709	Actor effect (BCR-Q)	− 0.174	− 0.306	0.761	0.392	0.688	0.494
Partner effect (BCR-Q)	0.057	0.111	0.912	− 0.100	− 0.195	0.845	Partner effect BCR-Q)	− 0.346	− 0.584	0.561	− 0.773	− 1.302	0.197
Actor effect (PCS)	0.118	0.774	0.441	0.135	0.887	0.378	Actor effect (PCS)	0.020	0.128	0.898	0.116	0.716	0.476
Partner effect (PCS)	0.324	2.447	0.017*	0.330	2.495	0.015*	Partner effect (PCS)	0.286	1.974	0.052	0.370	2.551	0.013*
Practical barriers:							Perceived suitability:						
Actor effect (BCR-Q)	−1.295	−2.156	0.034*	− 0.065	− 0.109	0.914	Actor effect (BCR-Q)	− 0.130	− 0.212	0.833	− 0.614	− 0.996	0.322
Partner effect (BCR-Q)	−0.433	− 0.558	0.578	0.873	1.124	0.264	Partner effect (BCR-Q)	− 0.020	− 0.027	0.978	−0.989	− 1.307	0.195
Actor effect (PCS)	0.181	1.196	0.235	0.122	0.809	0.421	Actor effect (PCS)	0.113	0.754	0.453	0.146	0.974	0.333
Partner effect (PCS)	0.306	2.332	0.022*	0.312	2.376	0.020*	Partner effect (PCS)	0.263	1.945	0.055	0.303	2.238	0.028*

*APIM* Actor Partner Interdependence Model, *PCS* physical component score, *SF-12* Short-From 12 Health Survey, *B-IPQ* Brief Illness Perception Questionnaire, *BCR-Q* Beliefs about Cardiac Rehabilitation **p < 0.001; *p < 0.05

For mental health (outcome), the patient’s greater concerns about their illness and greater emotional effect (B-IPQ) predicted their poorer mental health at 6 months (i.e. *actor effects)* (Table 4). In addition, the caregiver’s higher score for treatment control i.e. whether they perceived the patient’s treatment as helpful predicted their better mental health at 6 months (i.e. *actor effect)* (Table 4). There were no other statistically significant *actor effects* of baseline illness perceptions (B-IPQ) on the mental health of patients or caregivers at 6 months follow-up; and also no *actor effects* of baseline beliefs about CR (BCR-Q) on the mental health of patients or caregivers at 6 months (Table 4).

**Table 4:** Illness perceptions and beliefs about cardiac rehabilitation as predictors of mental health outcome (APIM)

Effect	Patients			Caregivers			Effect	Patients			Caregivers		
MCS outcome	Beta	*t*	*p*	Beta	*t*	*p*	MCS outcome	Beta	*t*	*p*	Beta	*t*	*p*
**Illness perceptions**							**Illness perceptions**						
Total score:							Consequences:						
Actor effect (B-IPQ)	−0.105	−0.837	0.405	−0.139	−1.104	0.273	Actor effect (B-IPQ)	1.030	1.987	0.050	1.078	2.082	0.041*
Partner effect (B-IPQ)	−0.255	−1.834	0.070	−0.103	− 0.745	0.459	Partner effect (B-IPQ)	−0.338	− 0.685	0.495	−0.681	−1.337	0.172
Actor effect (MCS)	0.737	4.428	< 0.001**	− 0.284	− 1.708	0.092	Actor effect (MCS)	0.871	5.550	< 0.001**	−0.140	− 0.892	0.375
Partner effect (MCS)	0.377	4.103	< 0.001**	−0.143	− 1.553	0.124	Partner effect (MCS)	0.453	5.006	< 0.001**	−0.093	−1.027	0.308
Timeline:							Personal control:						
Actor effect (B-IPQ)	−0.129	−0.319	0.750	0.086	0.212	0.832	Actor effect (B-IPQ)	1.288	1.880	0.064	0.558	0.815	0.418
Partner effect (B-IPQ)	−1.010	−2.044	0.044*	− 0.917	− 1.857	0.067	Partner effect (B-IPQ)	0.464	0.909	0.366	0.258	0.507	0.614
Actor effect (MCS)	0.744	4.863	< 0.001**	− 0.228	− 1.494	0.137	Actor effect (MCS)	0.884	5.690	< 0.001**	− 0.115	− 0.745	0.458
Partner effect (MCS)	0.485	5.076	< 0.001**	− 0.644	− 0.674	0.502	Partner effect (MCS)	0.376	3.885	< 0.001**	− 0.187	− 1.935	0.057
Treatment control:							Identity:						
Actor effect (B-IPQ)	1.409	1.675	0.098	3.293	3.914	< 0.001**	Actor effect (B-IPQ)	−0.788	− 1.380	0.171	− 1.087	− 1.902	0.061
Partner effect (B-IPQ)	0.812	0.872	0.386	− 0.655	− 0.704	0.483	Partner effect (B-IPQ)	− 0.167	− 0.304	0.762	− 0.112	− 0.204	0.839
Actor effect (MCS)	0.818	5.923	< 0.001**	− 0.134	− 0.974	0.333	Actor effect (MCS)	0.852	5.383	< 0.001**	− 0.109	− 0691	0.492
Partner effect (MCS)	0.386	4.591	< 0.001**	− 0.144	− 1.719	0.089	Partner effect (MCS)	0.414	4.549	< 0.001**	− 0.126	− 1.338	0.169
Illness concern:							Coherence:						
Actor effect (B-IPQ)	− 0.973	− 2.164	0.034*	−0.238	−0.530	0.597	Actor effect (B-IPQ)	0.048	0.052	0.959	1.812	1.958	0.054
Partner effect (B-IPQ)	− 1.356	− 2.360	0.021*	− 0.236	− 0.412	0.682	Partner effect (B-IPQ)	0.248	0.271	0.787	0.222	0.242	0.809
Actor effect (MCS)	0.802	5.474	< 0.011*	− 0.167	− 1.142	0.257	Actor effect (MCS)	0.827	5.235	< 0.001**	− 0.124	− 0.787	0.434
Partner effect (MCS)	0.391	4.232	< 0.001**	− 0.084	− 0.919	0.361	Partner effect (MCS)	0.374	3.584	< 0.001**	− 0.136	− 1.310	0.194
Emotional response:													
Actor effect (B-IPQ)	−1.278	− 2.834	0.006**	−0.876	− 1.944	0.055							
Partner effect (B-IPQ)	0.100	− 0.229	0.820	− 0.409	− 0.932	0.354							
Actor effect (MCS)	0.737	4.845	0.001**	− 0.197	− 1.298	0.198							
Partner effect (MCS)	0.447	5.016	< 0.001**	− 0.084	− 0.951	0.344							
**Beliefs about CR**							**Beliefs about CR**						
Necessity:							Exercise concern:						
Actor effect (BCR-Q)	−0.655	−1.066	0.290	1.127	1.833	0.071	Actor effect (BCR-Q)	−0.809	−1.212	0.229	0.811	1.214	0.228
Partner effect (BCR-Q)	0.295	0.512	0.610	0.282	0.489	0.626	Partner effect BCR-Q)	−0.674	−1.006	0.317	−0.427	− 0.638	0.525
Actor effect (MCS)	0.770	4.864	< 0.001**	− 0.054	−0.345	0.731	Actor effect (MCS)	0.719	4.158	< 0.001**	− 0.056	−0.327	0.745
Partner effect (MCS)	− 0.116	− 1.279	0.205	0.413	4.545	< 0.001**	Partner effect (MCS)	−0.117	− 1.248	0.216	0.389	4.133	< 0.001**
Practical barriers:							Perceived suitability:						
Actor effect (BCR-Q)	−1.150	−1.575	0.119	−0.464	−0.636	0.527	Actor effect (BCR-Q)	− 0.184	−0.255	0.800	−0.108	− 0.180	0.881
Partner effect BCR-Q)	−1.649	− 1.907	0.060	−0.385	− 0.446	0.657	Partner effect (BCR-Q)	−0.267	− 0.293	0.770	−0.016	− 0.018	0.986
Actor effect (MCS)	0.753	4.704	< 0.001**	− 0.141	−0.882	0.380	Actor effect (MCS)	0.812	5.140	< 0.001**	− 0.166	−1.052	0.296
Partner effect (MCS)	−0.886	− 0.963	0.338	0.442	4.802	< 0.001**	Partner effect (MCS)	−0.118	− 1.168	0.247	0.432	4.265	< 0.001**

*APIM* Actor Partner Interdependence Model, *MCS* mental component score, *SF-12* Short-From 12 Health Survey, *B-IPQ* Brief Illness Perception Questionnaire, *BCR-Q* Beliefs about Cardiac Rehabilitation **p < 0.001; *p < 0.05

**Fig. 2:**
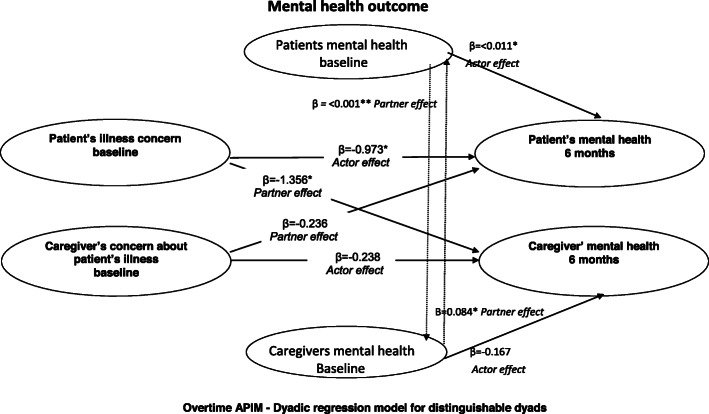
Results for the *actor* and *partner* effects of patient’s illness concern and caregiver’s concern about patient’s illness on the patient’s and caregiver’s mental health (SF-12) at 6 months; APIM: Actor-Partner Interdependence Model. *p < 0.05; ** *p* < 0.001

There was a statistically significant *partner effect* of timeline (B-IPQ) on the mental health (outcome) of caregivers. This indicated that the patient’s higher score for timeline i.e. greater duration of illness predicted the caregiver’s poorer mental health at 6 months follow-up (Table 4). Also, the patient’s higher score for illness concerns (B-IPQ) i.e. greater concerns about illness predicted the caregiver’s poorer mental health at 6 months *(*i.e. *partner effect)* (Table 4, Fig. 2). No other statistically significant *partner effects* were found for the individual’s (i.e., the patient’s and caregiver’s) illness perceptions at baseline impacting his or her partner’s mental health at 6 months follow-up; and also no *partner effects* of baseline beliefs about CR (BCR-Q) on the mental health of patients and caregivers at 6 months follow-up (Table 4).

There were however statistically significant *partner effects* of the patients’ and caregivers’ baseline physical health on the physical health (outcome) of the dyad at 6 months (Table 3). Also, there were statistically significant *actor and partner effects* of the patients’ baseline mental health on the mental health (outcome) of the dyads at 6 months (Table 4).

## Discussion

To our knowledge this is the first study to conduct longitudinal dyadic analysis with the aim of understanding the relationships between patients’ illness perceptions (and caregivers’ perceptions of the patient’s illness) and beliefs about CR upon entry to a CR programme and perceived physical and mental health (outcomes) at 6 months follow-up. Prior research has assessed patients’ illness perceptions (B-IPQ) [11, 25], and spouse’s perceptions of the patient’s illness [28], though data were not analysed using the APIM and different outcomes were evaluated. Further, while patients’ beliefs about CR and the relationship between such beliefs and CR attendance have been explored [29, 45], caregivers’ beliefs about CR using the BCR-Q have not been examined. Thus, the present study extends the body of knowledge on illness perceptions and beliefs about CR on the physical and mental health (outcomes) of CAD patient-caregiver dyads, using the APIM. Previous studies with cardiac patient-caregiver dyads have mostly employed the APIM to examine cross-sectional data [40–42, 46, 47], which meant that the direction of causality of associations could not be determined.

The first aim of the study was to assess differences in illness perceptions, beliefs about CR and physical and mental health between patients with CAD and their family caregivers upon entry to a CR program and 6 months later. We found that the caregivers had significantly higher scores for illness concerns (individual item, B-IPQ) at baseline and 6 months than the patients, suggesting they perceived a more threatening (negative) view of the patient’s illness. Prior research has highlighted the experience of a heart attack can be more distressing for patients’ relatives than for the patients themselves [48]. The other patient and caregiver B-IPQ (individual items), however, were not statistically significantly different. Overall, the patients’ mean scores for illness perceptions (B-IPQ) were similar to those of Broadbent et al. [25]. For caregivers, the mean scores for consequences, identity and coherence (B-IPQ) (individual items) were higher than those observed by Broadbent et al. [28], suggesting they perceived a greater impact of the illness on the patient’s life and more severe symptoms, but better coherence. Our finding for overall similarity in the patients’ and caregivers’ illness perceptions (individual items, B-IPQ) is consistent with prior research [49–51]. One explanation for this similarity may lie with its links to the theory of self-regulation which argues that the beliefs individuals hold about their illness and treatment are central to how they evaluate the effect of the illness on their lives [52, 53].

Our results for differences in the total scores for the B-IPQ indicate that the caregivers had higher scores than the patients at 6 months. The patients’ B-IPQ (total score) at baseline was comparable with those of Blair et al. [5], for attenders at CR. Several studies have examined the relationship between patients’ illness perceptions and behavioural outcomes, including attendance at CR [26, 30, 54], illness perceptions and quality of life [15, 25, 55–58], and whether an illness perception intervention reduces anxiety in spouses of MI patients [11, 28]. However, there remains a paucity of evidence on illness perceptions and physical and mental health (outcomes) in CAD patient-caregiver dyads.

For beliefs about CR (BCR-Q), there were no statistically significant differences between the patients and caregivers for necessity, exercise concerns, practical barriers or perceived suitability. Other investigators have examined patients’ beliefs about CR using the BCR-Q, alongside illness perceptions to predict attendance at CR but only necessity was really discussed [59]. Earlier research by Cooper et al. [45], identified that necessity and perceived suitability were positively related to CR attendance whilst practical barriers and concerns about exercise were associated with poorer attendance, however physical and mental health outcomes were not identified. Our results suggest there is still room for improvement in the patients’ and caregiver’s beliefs about CR. If patients do not believe in the benefits of CR they may be less likely continue participating [15, 29]. Therefore, incorrect beliefs about CR need to be targeted as soon as possible as part of the CR process and physical and mental health (outcomes) examined. Compared to Cooper et al. [29], the patients’ scores for concerns about exercise, barriers to CR and perceived suitability were lower (i.e. less positive) but higher (i.e. more positive) for necessity. For caregivers, no previous studies were found that used the BCR-Q for comparison of our results.

Our results for differences in mental health (outcomes) between the patients and caregivers revealed their scores were similar; a finding which is consistent with prior research [40, 60, 61]. The EUROACTION study also showed that couples often share similar ratings of health-related quality of life [49]. Our patients’ physical health was poorer at baseline and 6 months, compared to the caregivers and it remained below the population average of 50 at 6 months [62, 63]. This poorer physical health in patients may be attributed to their initial physical limitation and angina symptoms although one would expect this to improve with CR [1, 64]. Our patients’ CR programme lasted 12 weeks; it may be that their physical health improved during CR but deteriorated again on completion of the programme [65]. Several models of cardiac rehabilitation have been identified in the literature with various compliance rates. These appear particularly low for older adults. However, recently Campo et al. reported an early, low cost model with very promising results in terms of physical performance after myocardial infarction and quality of life [66, 67]. Our findings for caregivers are consistent with Ebbesen et al. [68], who identified that caregivers often have poor emotional and physical health-related quality of life. This is important to identify because if the caregivers’ health is poor this may be detrimental to patient’s health and recovery [60].

The second aim of the study was to examine whether the patient’s illness perceptions (and caregiver’s perceptions of the patient’s illness) and beliefs about CR at baseline predicted their own and their partner’s physical and mental health (outcomes) at 6 months follow-up. Our results revealed that the patients’ physical health at 6 months was predicted by their level of coherence (B-IPQ) and practical barriers to CR (BCR-Q) upon entry to a CR programme (i.e. *actor effects*). Consistent with our findings, other studies have found a positive correlation between coherence and physical health but in simple regression [15]. Consistent with Cooper et al. [29, 45], and Herber et al. [69], we identified that the patients’ perceived significant barriers to CR, but quality of life was not assessed in these studies. We identified the patient’s perceptions of control (B-IPQ) did not significantly predict their physical health (i.e. *no actor effect*), a finding which is similar to Janssen et al. [15], who found no relation between perceptions of control and physical health. For caregivers, level of coherence did not significantly predict their physical health (i.e. no *actor effect*). This finding is contrary to Broadbent et al. [28], however, they did not analyse dyadic data using the APIM. Several other studies have examined illness perceptions and quality of life in CAD but only patients’ quality of life was assessed [15, 26, 27, 55, 58].

Our results revealed there were no statistically significant *partner effects* of illness perceptions and beliefs about CR on physical health i.e. the patient’s illness perceptions and beliefs about CR did not predict their partner’s (i.e. the caregiver’s) physical health (outcome) at 6 months. Similarly, the caregiver’s perception of the patient’s illness and beliefs about CR did not predict their partner’s (i.e. the patient’s) physical health (outcome) at 6 months (no *partner effects*). Prior longitudinal research with cancer patient-caregiver dyads has similarly shown negative findings for predictors of physical health (outcomes) [70]. A noteworthy finding in our research was that both the patients’ and caregivers’ physical health at baseline impacted negatively on the physical health of the dyad at 6 months. No longitudinal studies of cardiac patients-caregiver dyads were found for comparison of our results.

The results revealed that the patient’s poorer mental health (outcome) at 6 months was predicted by their greater concerns about illness and emotional response (B-IPQ) upon entry to a CR programme (i.e. *actor effects*). These associations between illness concerns and emotional response have been found in prior research [15, 25], and meta-analysis of the B-IPQ [56]. It was a surprise finding the patient’s beliefs about CR (BCR-Q) did not significantly predict their mental health (outcome) at 6 months (i.e. no *actor effects*). Evidence from meta-analyses of randomised controlled trials show that CR programmes facilitate physical and psychological recovery following acute cardiac events [1, 64]. Our dyadic analysis revealed statistically significant *actor effects* of the caregiver’s baseline perceptions of treatment control and consequences of illness (B-IPQ) on their mental health at 6 months. These results highlight the potential benefits of promoting positive illness perceptions on the mental health (outcomes) of caregivers.

Our results showed there were statistically significant *partner effects* of timeline and illness concern on the mental health (outcomes) of caregivers i.e. patient’s higher scores for timeline (greater duration of illness) and greater illness concern significantly predicted the caregiver’s poorer mental health at 6 months. This suggests the caregiver’s mental health may be particularly vulnerable to the illness perceptions of their partner’s i.e. the patient. No APIM studies of illness perception in CAD patient-caregiver dyads were found for comparison of our results. Previous cross-sectional studies using the APIM have shown *partner effects* of self-esteem and optimism on depressive symptoms of spousal caregivers [46], and a *partner effect* of informational/emotional support on the mental health of caregivers/partners [42]. In addition, in this study we identified the patients’ mental health at baseline impacted on the mental health of the dyad at 6 months i.e. significant *actor* and *partner effects*. Prior longitudinal research with cancer patient-caregiver dyads has shown a *partner effect* of mental health i.e. the patients’ mental health at 6 months was associated with the caregiver’s mental health at 3 months [70].

### Strengths and limitations

There are strengths and limitations to the study. Firstly, this was a relatively small sample of patients and caregivers recruited from one CR centre. We did not have enough information on the non-respondents to decide whether they would have differed in terms of outcome from the rest of the sample or not. Nevertheless, the study aimed to be representative of the wider UK CR population as patients were selected from a standard hospital-based CR service. We requested that caregivers and patients completed the questionnaire separately from each other. The researcher had no way of ensuring that the caregiver did not discuss their answers with the patient. This may be considered a limitation of the study. Strengths of the study lie in its recruitment of patient-caregiver dyads, in its longitudinal design and in the selection of antecedent variables i.e. illness perceptions and beliefs about CR that to our knowledge have not been used before in dyadic analysis (using the APIM), to predict patients and caregivers physical and mental health outcomes. Secondly, we collected data at two time points i.e. upon initial attendance at the CR program (baseline) and at 6 months follow-up. The inclusion of 3 or more follow-up times may have been useful in identifying the patterns of change overtime. This was not feasible given our small sample size and potential for dropouts. Thirdly, we did not include causal attributions as part of the dyadic analysis but we computed both the B-IPQ (total scores) and individual items. This is important because summing items may lose information about the perceptions most strongly linked to outcomes [56].

### Implications for practice

There are several implications resulting from the study findings. Firstly, because the patient’s perceived timeline and illness concerns (B-IPQ) at baseline predicted the caregiver’s poorer mental health at 6 months these particular illness perceptions need to be explored early i.e. possibly on the first hospital visit from the CR specialist to promote caregivers’ better mental health and enable their ongoing support of the patient. Secondly, the patient’s negative illness concerns and greater emotional response (B-IPQ) at baseline predicted their poorer mental health at 6 months. This highlights the need to further explore with the patient their specific illness concerns and to provide emotional support early in the rehabilitation process. More research is needed to replicate our study findings and to decipher which illness perceptions and beliefs about CR have more influence on the individual and the dyad over time and to target these appropriately. Fourthly, intervention design should include caregivers, addressing their specific beliefs and concerns. Although CR programmes often include caregivers in educational sessions, they receive less information than patients and the focus is often on improving patient outcomes. More longitudinal research is needed with a larger sample of patient-caregiver dyads in CAD to explore interpersonal relationships and dyadic outcomes.

## Conclusions

Overall, the patients and caregivers in this study had similar scores for illness perceptions and beliefs about CR which prevailed over time. The patient’s perceptions of timeline and illness concern (B IPQ) upon entry to a CR programme are especially important for they predicted the mental health of the caregiver at 6 months (i.e. *partner effects*). The patient’s illness perceptions (and caregiver’s perception of the patient’s illness) and beliefs about CR at baseline predicted their own physical and mental health (outcomes) at 6 months (i.e. *actor effects*). Both the patients’ and caregiver’s negative illness perceptions and beliefs about CR need to be addressed early as part of CR to help improve physical and mental health (outcomes) at 6 months. Interventions need to be tested that focus on specific illness perceptions and beliefs about CR, targeting both the individual and the dyad, to help improve patient and caregiver physical and mental health outcomes.

## Data Availability

The datasets used and/or analysed during the current study are available from the corresponding author on reasonable request.

## Abbreviations

CR: Cardiac rehabilitation
B-IPQ: Brief Illness Perception questionnaire
BCR-Q: Beliefs about Cardiac Rehabilitation questionnaire
Dyads: Patient-caregiver pairs
APIM: Actor–Partner Interdependence Model
CAD: Coronary artery disease
SF-12v2: Short-Form 12 Health Survey
PCS: Physical component score
MCS: Mental component score
LVEF: Left ventricular ejection fraction
SPSS: Statistical Package for Social Scientists
NSTEMI: Non-ST elevation myocardial infarction
STEMI: ST elevation myocardial infarction
MI: Myocardial infarction
